# Aggregation in the spotlight

**DOI:** 10.7554/eLife.73586

**Published:** 2021-10-12

**Authors:** Zihao Wang, Miranda Collier, Justin Benesch

**Affiliations:** 1 Department of Chemistry, Oxford University Oxford United Kingdom; 2 Department of Biology, Stanford University Stanford United States

**Keywords:** molecular condensates, RRM, chaperones, time-resolved quantitative XL-MS, FUS, aging, Human

## Abstract

New findings clarify apparently conflicting results about how molecular agents that preserve protein integrity prevent harmful, dense aggregates from forming.

**Related research article** Boczek EE, Fürsch J, Niedermeier ML, Jawerth L, Jahnel M, Ruer-Gruß M, Kammer KM, Heid P, Mediani L, Wang J, Yan X, Pozniakovski A, Poser I, Mateju D, Hubatsch L, Carra S, Simon Alberti D, Hyman AA, Stengel F. 2021. HspB8 prevents aberrant phase transitions of FUS by chaperoning its folded RNA binding domain. *eLife* **10**:e69377. doi: 10.7554/eLife.69377

From bacteria to plants to humans, all organisms carry a group of molecular chaperones dedicated to helping cells withstand various stressors such as heat, drought, aging or injury. These small heat shock proteins (or sHsps) help cells adapt to stress by associating with damaged proteins. Without this help, the proteins may form insoluble, harmful aggregates such as amyloid fibrils, which are associated with aging and disease.

Yet, seemingly conflicting evidence has made it difficult to understand how sHsps work. Traditionally, they are thought to prevent proteins from aggregating; however, in some cases sHsps seem to actually promote protein accumulation (Haslbeck and Vierling, 2015; Escusa-Toret et al., 2013; Mymrikov et al., 2017; Walther et al., 2015). Intuitively inconsistent, this picture suggests that the sHsp mechanism is yet to be fully pieced together (Mogk et al., 2019). Now, in eLife, Florian Stengel and colleagues from institutions in Germany and Italy – including Edgar Boczek and Julius Fürsch as first co-authors – report how this apparent contradiction might be reconciled (Boczek et al., 2021).

The team built on observations from 35 years ago that sHsps are found in membrane-free structures that often form in cells under stress (Collier and Schlesinger, 1986). Now understood to be droplet-like, these ‘condensates’ store select proteins and other biological molecules, keeping them safely huddled together until the cell recovers (Wallace et al., 2015). But proteins being present in such high concentrations can also be dangerous. If they become unstable and misfold in the condensates, there is an increased risk that they will start to form amyloid fibrils (Alberti et al., 2017; Zbinden et al., 2020). In this perilous environment, sHsps may be key to protect protein stability.

This possibility inspired Boczek et al. to study how a sHsp present in the human brain and muscles (HspB8, also known as HSP22) interacts with an RNA-binding protein that readily enters condensates and can form aggregates. Mutations within this protein – which is known as FUS, short for Fused in sarcoma – can lead to the motor neuron disease known as amyotrophic lateral sclerosis (ALS).

Boczek et al. developed a new approach that allowed them to map how the two proteins interact under different conditions. This was accomplished by combining chemical crosslinking (where compounds are used to permanently link proteins that briefly interact) and mass spectrometry (which gives a ‘snapshot’ of proteins by measuring their parts). Linking the proteins before collecting the snapshots allowed for structural insight, enabling the team to determine which parts of HspB8 and FUS were in contact inside and outside of the condensates.

This approach showed that the presence of HspB8 inside condensates containing FUS slowed down the hardening of the droplets by preventing amyloid fibrils from forming. By analysing crosslinks, Boczek et al. were able to identify which regions of the proteins are responsible for this protective mechanism. This revealed that structural changes in the segment of FUS which allows the protein to recognise RNA underlie hardening. However, when this motif is bound by a section of HspB8 known as the α-crystallin domain — an interaction that only occurs within condensates — FUS cannot turn into aggregates. In turn, a mutant version of HspB8 where the α-crystallin domain is altered was not able to protect FUS condensates against amyloid formation. This mutation in HspB8 is associated with a distal motor neuropathy, which, like amyotrophic lateral sclerosis, causes muscle weakness.

Further results showed that the disordered N-terminal domain of HspB8 was necessary for its recruitment to FUS condensates, a mechanism also observed in a related chaperone called HspB1 (Liu et al., 2020). Comparing how HspB8 and HspB1 interact with FUS helps to better understand how these chaperones work in general. In particular, unlike HspB8 which functions inside condensates, HspB1 can protect FUS both inside (if the chaperone carries a chemical modification) and outside of these structures (Liu et al., 2020). HspB8 and HspB1 are sometimes present in the same small groups of proteins, raising the question of how co-operation amongst sHsps benefits the cell (Sun et al., 2004).

A coherent picture of the sHsp mechanism is therefore gradually emerging, aided substantially by the findings of Boczek et al. It appears that, whether alone or together, sHsps can ensure that proteins do not form insoluble structures during stress. The chaperones may seem to both promote and prevent protein aggregation, but this apparent contradiction can be reconciled by considering that what researchers previously called ‘aggregation’ in fact encompasses different ways that molecules can come together. When required by the cell, sHsps help to gather proteins into condensates in which they are more densely packed than before but remain dynamic. There, the chaperones prevent proteins from forming harmful aggregates such as amyloid fibrils (Figure 1). Obtaining data on sHsp interactions in vivo across the tree of life is now needed to confirm whether this mechanism occurs in the diverse organisms that host these enigmatic chaperones.

**Figure 1.**
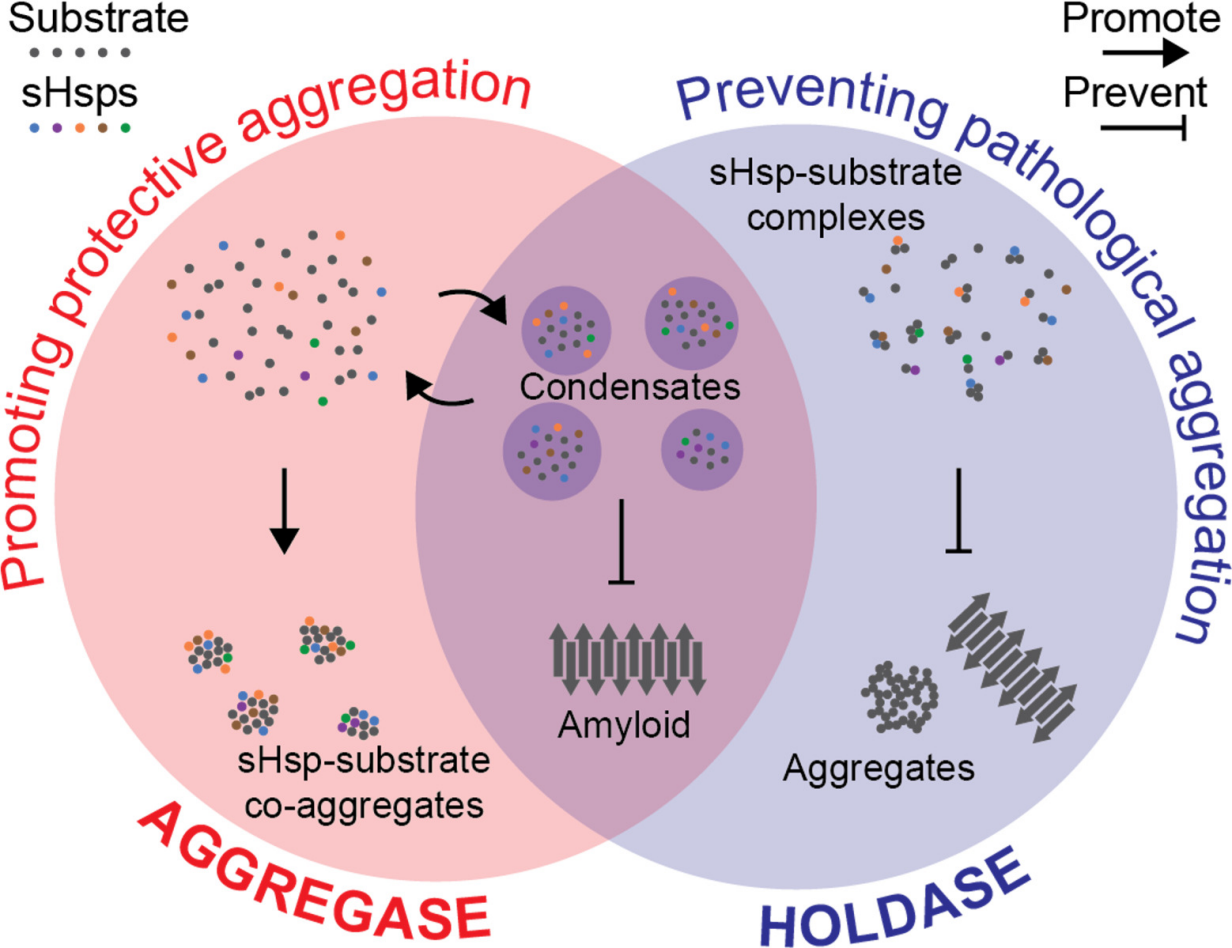
Hypothesis for a coherent mechanism of sHsp chaperone function. Chaperones known as sHsps (colourful dots) can bind to proteins destabilised under stress conditions (‘substrates’; grey dots). They either encourage other proteins to gather with them in large protective complexes known as co-aggregates (‘aggregase’ activity; red, left); or they keep the proteins they bind apart from each other (‘holdase’ activity; blue, right) to stop these substrates from transitioning into toxic aggregates such as amyloid fibrils (grey arrows). These apparently incompatible models of chaperones’ activity can be reconciled by considering that they both aim to stop destabilised proteins from coming together in specific ways that are harmful to the cell. Sometimes, this aim is achieved by forming reversible liquid condensates requiring both aspects of sHsp function: there, the chaperones help to sequester the proteins and prevent them from becoming amyloid fibrils.
